# Determining the GmRIN4 Requirements of the Soybean Disease Resistance Proteins Rpg1b and Rpg1r Using a *Nicotiana glutinosa*-Based Agroinfiltration System

**DOI:** 10.1371/journal.pone.0108159

**Published:** 2014-09-22

**Authors:** Ryan Kessens, Tom Ashfield, Sang Hee Kim, Roger W. Innes

**Affiliations:** Department of Biology, Indiana University, Bloomington, Indiana, United States of America; UMBC, United States of America

## Abstract

*Rpg1b* and *Rpg1r* are soybean disease resistance (*R*) genes responsible for conferring resistance to *Pseudomonas syringae* strains expressing the effectors AvrB and AvrRpm1, respectively. The study of these cloned genes would be greatly facilitated by the availability of a suitable transient expression system. The commonly used *Niciotiana benthamiana*-based system is not suitable for studying *Rpg1b* and *Rpg1r* function, however, because expression of AvrB or AvrRpm1 alone induces a hypersensitive response (HR), indicating that *N. benthamiana* contains endogenous *R* genes that recognize these effectors. To identify a suitable alternative host for transient expression assays, we screened 13 species of *Nicotiana* along with 11 accessions of *N. tabacum* for lack of response to transient expression of *AvrB* and *AvrRpm1*. We found that *N. glutinosa* did not respond to either effector and was readily transformable as determined by transient expression of β-glucuronidase. Using this system, we determined that *Rpg1b*-mediated HR in *N. glutinosa* required co-expression of *avrB* and a soybean ortholog of the Arabidopsis *RIN4* gene. All four soybean *RIN4* orthologs tested worked in the assay. In contrast, Rpg1r did not require co-expression of a soybean *RIN4* ortholog to recognize AvrRpm1, but recognition was suppressed by co-expression with AvrRpt2. These observations suggest that an endogenous *RIN4* gene in *N. glutinosa* can substitute for the soybean *RIN4* ortholog in the recognition of AvrRpm1 by Rpg1r.

## Introduction

Effector-triggered immunity (ETI) depends on the expression of disease resistance (*R*) genes, the majority of which encode nucleotide binding-leucine rich repeat (NB-LRR) proteins. NB-LRR proteins have been shown to detect effectors by both direct and indirect mechanisms [1]. Indirect recognition requires an intermediate protein that serves as a substrate for the effector. These intermediate proteins are targeted by effectors and can be modified in ways such as proteolytic cleavage or phosphorylation. This allows R proteins to indirectly detect the presence of effectors by monitoring the status of the effector targets [2].

AvrB and AvrRpm1 are two effectors found in certain strains of *Pseudomonas syringae* [3], [4], the causative agent of soybean bacterial blight and related diseases in other plant species. In *Arabidopsis thaliana,* the *R* gene *RPM1* confers resistance to bacteria expressing both of these effectors, but only if a functional copy of *RIN4* is also present in the genome [5], [6]. This is considered a classic example of indirect recognition. The R protein (RPM1) guards the effector target (RIN4), which is modified in the presence of both AvrB and AvrRpm1 [6]. Both of these effectors are thought to modify RIN4 through phosphorylation, but whether this is direct or indirect remains unclear [7] [8]. Phosphorylation of RIN4 is detected by RPM1, which then initiates a signaling cascade that leads to a form of programmed cell death known as the hypersensitive response (HR) that inhibits the spread of the invading pathogen [9].

While recognition of AvrB and AvrRpm1 in soybean likely has similarities to that in Arabidopsis, there are at least two key differences. Unlike Arabidopsis, which requires just one *R* gene to confer resistance to both AvrB and AvrRpm1, soybean requires two *R* genes. These *R* genes are *Rpg1b* and *Rpg1r*, which confer resistance to AvrB and AvrRpm1, respectively [10]. Another difference is that the Arabidopsis genome only encodes one *RIN4* gene while soybean contains four *RIN4*-homologues (*GmRIN4a, GmRIN4b, GmRIN4c,* and Gm*RIN4d*) [11]. At least two of these family members, *GmRIN4a* and *GmRIN4b*, appear to be necessary for *Rpg1b* to confer resistance to AvrB [12].

Several *R* gene recognition systems have been reconstituted in *Nicotiana benthamiana* by infiltrating leaves with a mixture of *Agrobacterium tumefaciens* strains that transfer genes coding for an effector, R protein, and any intermediate protein(s) that might be necessary for an R protein to recognize the effector [13]-[15]. If an *R* gene confers resistance to a particular effector, it will initiate a signaling cascade leading to HR, which can be observed on plant leaves as brown discoloration and/or leaf collapse. While *N. benthamiana* is a useful transient system for investigating many *R* genes in this manner, it has limitations for the study of AvrB and AvrRpm1-specific *R* genes. Specifically, *N. benthamiana* contains endogenous *R* gene(s) able to detect these pathogen effectors. This makes it difficult to use *N. benthamiana* to study *R* genes from other species that recognize AvrB and/or AvrRpm1 because expression of these effectors alone triggers HR. While this can be partially mitigated by careful titration of the density at which the Agrobacterium strains are infiltrated [8], it would be useful to identify an alternative plant species for these experiments that does not respond to AvrB and AvrRpm1 [8], [16].

The goal of this study was to identify an alternative species to *N. benthamiana* for transient expression studies involving soybean Rpg1b and Rpg1r and to determine the *Gm*RIN4 requirements of each of these R proteins. An ideal species would have many of the characteristics that make *N. benthamiana* a good system such as ease of injection and high-transformation efficiency, but would not display signs of HR when *AvrB* and *AvrRpm1* were expressed alone. To achieve this goal, we screened *Nicotiana* germplasm, including 13 distinct species and 11 accessions of *N. tabacum*, for their response to transiently expressed *AvrB* and *AvrRpm1*. The transformation efficiency of each species was also assessed using a *GUS* reporter gene to ensure that lack of HR was not simply due to low levels of gene expression. The well-studied *RPS5*-mediated HR pathway was reconstituted in the most promising genotype (*N. glutinosa*) to test its efficacy for reconstructing an *R* gene pathway. This was accomplished by co-expressing the *P. syringae* effector *AvrPphB*, its target *PBS1*, and the *R* gene *RPS5* [17]. The final step was to co-express each of the soybean *R* genes with their corresponding effectors and one or more of the *GmRIN4*s to determine which, if any, *Gm*RIN4s were required by either R protein to detect its corresponding effector.

## Results

### Screening *Nicotiana* germplasm for accessions in which AvrB and AvrRpm1 do not trigger an HR

To identify *Nicotiana* species/accessions that do not respond to transient expression of the effectors *AvrB* and *AvrRpm1* with an HR, we used a two-step approach. Thirteen distinct species of *Nicotiana* and 11 accessions of *N. tabacum* were visually assessed for signs of effector recognition upon transient expression of *AvrB* or *AvrRpm1*. Effector recognition was determined by looking for morphological changes on plant leaves expressing either effector. These changes in morphology included brown discoloration on the adaxial and abaxial surface of leaves, a “shiny” phenotype on the abaxial surface, or full leaf collapse (Fig. S1). As a negative control, each plant was infiltrated with *Agrobacterium* carrying a plasmid with a *GUS* reporter gene. One leaf on each plant was transformed with *GUS* and compared to another leaf on the same plant expressing *AvrB* on one half and *AvrRpm1* on the other. Lack of effector recognition was assumed when the response to infiltration with the AvrB and AvrRpm1-containing strains was no stronger than the response to transformation with *GUS*. Six species of *Nicotiana* did not respond to either effector: *N. alata, N. glutinosa, N. knightiana, N. nudicaulis, N. rotundifolia,* and *N. tomentosiformis* (Table 1). All 11 accessions of *N. tabacum* screened exhibited signs of HR when expressing at least one of the effectors (Table 1).

**Table 1:** *Nicotiana* accessions used in this study and their responses to AvrB and AvrRpm1.

		Response to AvrB		Response to AvrRpm1	
PI number	Type	Response Characteristics	Effector-dependent response	Response Characteristics	Effector-dependent response
***N. tabacum*** ** accessions**					
552452	Maryland	s, b	+	s	+
404956	Oriental	s, b	+	s, b	+
378072	Oriental	s, b, lc	+	s, b	+
405603	Oriental	b	+	s	+
292205	Cigar filler	b	+	s	+
405604	Cigar filler	s, b	+	s, b	+
552348	Cigar binder	s, b	+	s	+
552619	Cigar wrapper	s, b, lc	+	s, b	+
552453	Flume cured	b, lc	+	s, b	+
543792	Burley	b, lc	+	b	+
551280	Burley	b, lc	+	b, lc	+
***Nicotiana*** ** species**					
42337	*N. langsdorffii*	b	+	nr	-
555531	*N. longiflora*	s, b	+	s, b	+
241768	*N. glutinosa*	s	-	s	-
555553	*N. rotundifolia*	nr	-	nr	-
555527	*N. knightiana*	nr	-	nr	-
555570	*N. sylvestris*	nr	-	s, b	+
555554	*N. rustica*	s, b	+	s, b	+
555552	*N. repanda*	s, b	+	nr	-
42334	*N. alata*	nr	-	nr	-
503323	*N. debneyi*	b	+	b	+
555540	*N. nudicaulis*	nr	-	nr	-
555572	*N. tomentosiformis*	nr	-	nr	-

A (+) sign indicates that leaves expressing a given effector gave a stronger response than leaves expressing *GUS*, while a (-) sign indicates there was no difference between leaves expressing an effector and those expressing *GUS.* The observed morphologies in response to *Agrobacterium*-mediated transformation were leaf browning (b), shininess on the abaxial surface (s), leaf collapse (lc), and no response (nr). See Figure S1 for photographs of phenotypes. Each plant species/accession was tested at least 3 times with similar results.

Before proceeding, we eliminated *N. alata* and *N. rotundifolia* as potential transient systems because of problems inherent to both species. *N. alata* displayed poor seed germination while *N. rotundifolia* was difficult to infiltrate with Agrobacterium. The transformation efficiency of the remaining species was determined to ensure that a lack of HR was not due to poor transgene expression. This was accomplished by quantifying β-glucuronidase activity in leaf tissue following infiltration with the Agrobacterium strain containing the *GUS* reporter gene. For comparison, *N. benthamiana* leaves were also transiently transformed with the *GUS*-containing strain, leaves harvested, and enzyme activity assayed in conjunction with each of the non-responding species. The results indicated that transgene expression levels in all of the species, including *N. benthamiana*, were quite variable between different individuals of the same genotype. This variation occurred even though all of the plants used in a given experiment were grown under the same conditions and great effort was taken to ensure conditions were consistent between experiments. Despite this variation, we found that *N. benthamiana* and *N. glutinosa* consistently yielded the highest *GUS* expression levels (Fig. S2). We also assayed the transformation efficiency of *N. knightiana* and *N. nudicaulis*, which consistently resulted in a GUS activity 5 to 10 fold lower than that observed in *N. benthamiana*.

### Reconstituting the *RPS5*, *Rpg1b*, and *Rpg1r*-mediated disease resistance pathways

**Figure 1:**
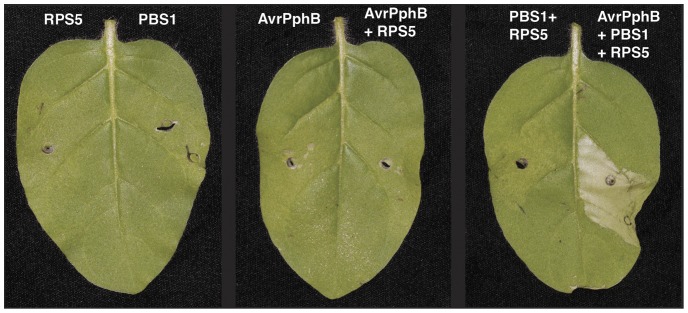
Reconstituting the *RPS5*-mediated defense pathway in *N. glutinosa*. The left and right side of each leaf were transiently transformed with the gene(s) listed above each image. Leaves were detached and photographed 24 hours after dexamethasone induction.

To assess whether *N. glutinosa* can be used as a transient system to reconstitute NB-LRR signaling pathways, we first tested the *RPS5* pathway from Arabidopsis, as this pathway has previously been successfully reconstituted in *N. benthamiana* [13]. RPS5 is an NB-LRR disease resistance protein from Arabidopsis that confers resistance to *P. syringae* strains expressing the effector gene *AvrPphB* [17]. Recognition of AvrPphB by RPS5 requires another Arabidopsis protein, PBS1, which is proteolytically cleaved by AvrPphB [18]. The N and C-terminal cleavage products of PBS1 bind to and activate RPS5 resulting in an HR [19]. We reconstituted the *RPS5*-mediated defense pathway by co-expressing *AvrPphB*, *PBS1,* and *RPS5* in *N. glutinosa*. As expected, strong leaf collapse was observed at the site of Agrobacterium infiltration when all three of the genes were expressed, but not when leaves lacked expression of any one component of the pathway (Fig. 1).

**Figure 2:**
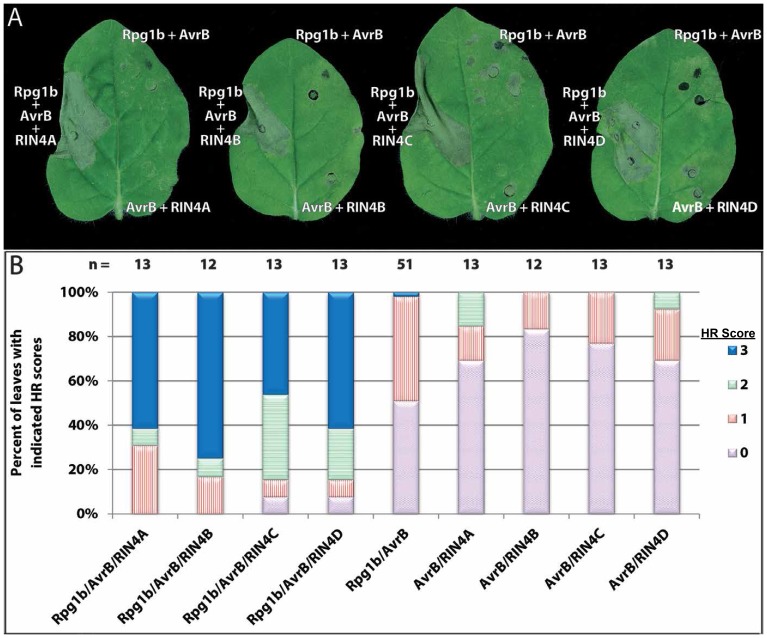
Reconstituting the *Rpg1b*-mediated defense pathway in *N. glutinosa*. (**A**) Activation of Rpg1b by AvrB requires co-expression of a *GmRIN4* gene. The images shown are of typical responses displayed by *N. glutinosa* leaves expressing the combination of genes labeled on each image. (**B**) Quantification of *Rpg1b*-mediated HR when co-expressed with various combinations of *AvrB* and *GmRIN4* genes. The strength of Rpg1b-mediated HR was determined by the extent of leaf collapse in the infiltrated area. Based on the extent of leaf collapse in the infiltrated area, plant leaves were categorized into 4 classes: 0 (no collapse); 1 (less than one third collapsed); 2 (one third to two thirds collapsed); 3 (greater than two thirds collapsed). Images were taken and plant leaves were scored approximately 2 days after transgene induction. The number of leaves infiltrated and scored for each combination (n) is listed above each bar. This experiment was repeated 3 times with similar results.

After successfully reconstituting the *RPS5* pathway, we investigated which components were necessary to reconstitute the *Rpg1b* pathway. This was accomplished by co-transforming *N. glutinosa* leaves with combinations of *AvrB*, *Rpg1b*, and the *GmRIN4*s. Leaves expressing a combination of *AvrB*, *Rpg1b*, and at least one of the *GmRIN4*s consistently gave a stronger response than leaves expressing *AvrB* and *Rpg1b* or *AvrB* and a *GmRIN4*. Figure 2a shows representative leaves expressing each combination, while Figure 2b shows an assessment of the strength of HR for each combination.

The finding that Rpg1b required co-expression of a GmRIN4 to detect AvrB raised the question of whether Rpg1r would similarly require a GmRIN4 to detect AvrRpm1. We have recently cloned Rpg1r (Genbank accession number KF958751; [20]). Transient overexpression of Rpg1r by itself in *N. glutinosa* induced a visible collapse, indicating that Rpg1r possesses autoactivity when overexpressed [20]. We found, however, that this autoactivity could be nearly eliminated by fusing super yellow fluorescent protein onto the C-terminus of Rpg1r [20]. Using this Rpg1r-sYFP construct, we were able to assess whether Rpg1r required co-expression of a GmRIN4 to induce HR in response to AvrRpm1. Unlike Rpg1b, co-expression of Rpg1r-sYFP and AvrRpm1 in the absence of GmRIN4 was sufficient to induce leaf collapse in *N. glutinosa* [20]. Importantly, co-expression of Rpg1r with AvrB in *N. glutinosa* did not induce HR regardless of whether a GmRIN4 was co-expressed, which established that the specificities of Rpg1b and Rpg1r are retained in this system [20]. Notably, co-expression of GmRIN4 with untagged Rpg1r did not suppress its autoactivity [20], indicating that Rpg1r autoactivity is not a consequence of activation of Rpg1r by loss of RIN4, as has been reported for the Arabidopsis RPS2 protein [21].

**Figure 3:**
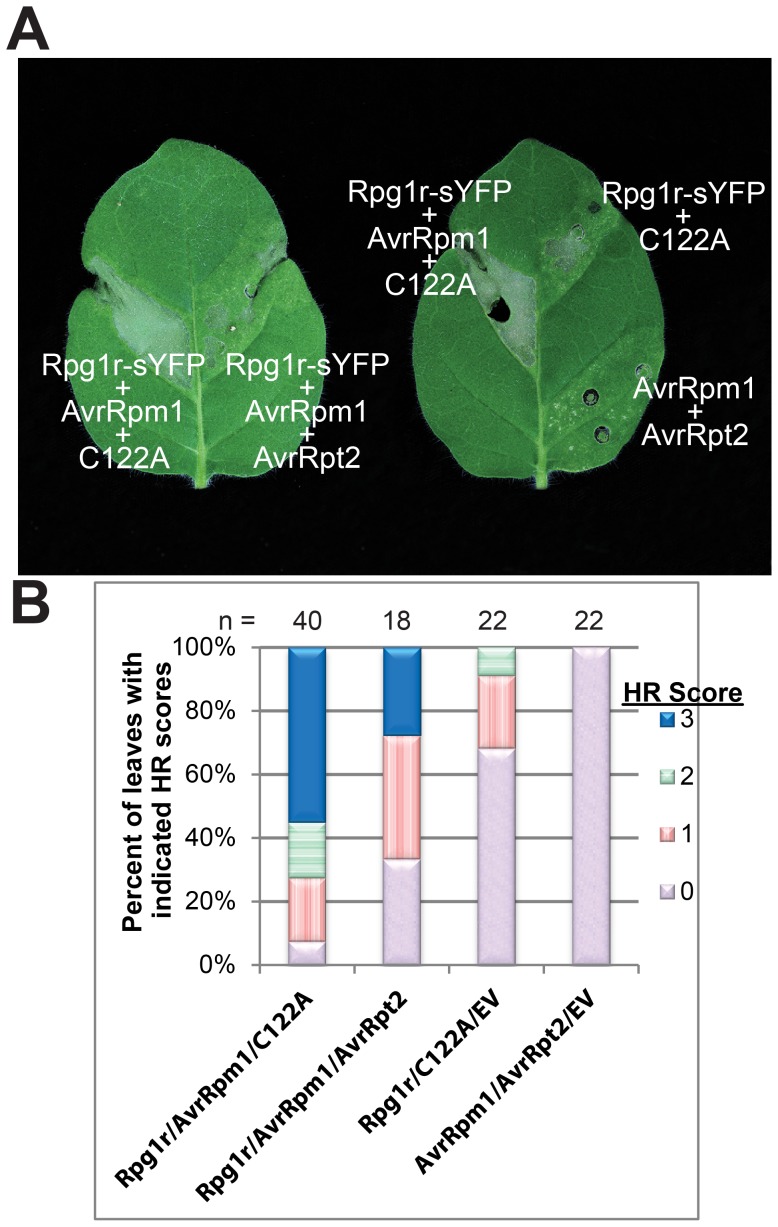
Reconstituting the *Rpg1r*-mediated defense pathway in *N. glutinosa*. (**A**) *Rpg1r*-*sYFP*-mediated HR does not require co-expression of a *GmRIN4* gene, but is suppressed by *avrRpt2*. The images shown are of typical responses displayed by *N. glutinosa* leaves expressing the combination of genes labeled on each image. (**B**) Quantification of *Rpg1r*-*sYFP* mediated HR. Responses were categorized as described in Figure 2. C122A indicates the protease inactive form of AvrRpt2. Images were taken and plant leaves were scored approximately 2 days after transgene induction. The number of leaves infiltrated and scored for each combination (n) is listed above each bar. This experiment was repeated 3 times with similar results.

Since Rpg1r did not require co-expression of a GmRIN4 to recognize AvrRpm1 in *N. glutinosa*, we hypothesized that it may be using an endogenous RIN4 protein for this purpose. To test this hypothesis, we employed the *P. syringae* effector AvrRpt2, which is a cysteine protease that has been shown to cleave Arabidopsis RIN4, leading to its degradation [22]. A BLAST search of the *N. benthamiana* genome using the Arabidopsis RIN4 amino acid sequence as the query revealed two predicted full length proteins with high sequence similarity and conserved AvrRpt2 cleavage sites [23] (Fig. S3). By expressing *AvrRpt2* under the constitutively active CaMV 35S promoter, we hoped to eliminate any endogenous RIN4 homologues in *N. glutinosa* before inducing the expression of *Rpg1r* and *AvrRpm1*, both of which were under the control of a DEX-inducible promoter. Co-expression of *AvrRpt2* with *Rpg1r* and *AvrRpm1* led to a reduction in the severity of HR, as indicated by reduced leaf collapse, compared to co-expression of the proteolytically inactive *AvrRpt2 (C122A)* mutant with *Rpg1r* and *AvrRpm1*. The images in Fig. 3a are representative of the typical responses displayed by leaves expressing each combination while Figure 3b is an assessment of the strength of HR for each combination. These data suggest that Rpg1r is employing an endogenous copy of RIN4 for recognition of AvrRpm1.

## Discussion

*P. syringae* strains have a wide host range and include pathovars such as *P. syringae* pv. *tabaci* that infect *Nicotiana* species. It is thus not surprising that *N. benthamiana* has evolved the ability to recognize specific *P. syringae* effector proteins. Individual *P. syringae* strains express numerous effectors, with great variation in specific effector repertoire between strains [24], [25]. This large effector complement is likely the result of a co-evolutionary arms race between *P. syringae* and its host plants. The goal of this study was to identify a *Nicotiana* species that lacked endogenous *R* genes with the ability to recognize *AvrB* and *AvrRpm1* expression and use this species to determine the *RIN4* requirements of *Rpg1b* and *Rpg1r*.

While *N. benthamiana* is widely used by plant biologists for transient gene expression, its ability to recognize the *P. syringae* effectors AvrB and AvrRpm1 makes it unsuitable for structure/function studies on the corresponding R proteins responsible for detecting these effectors. The benefits of *N. benthamiana* as a transient expression system, along with the great species diversity of *Nicotiana*, make this genus a good candidate for finding other species that could serve as suitable transient expression systems. While the six species highlighted in Table 1 did not respond to either effector in the initial screen, quantifying GUS transgene expression showed that a lack of a response in many of the species could be attributed to poor transformation efficiency. Even *N. glutinosa*, the species that gave the highest and most consistent transformation efficiency, gave variable results within and between experimental replicates. Important factors for obtaining efficient transformation included using young plants (∼4 weeks old), avoiding the youngest and oldest leaves (typically the 3rd and 4th true leaves were injected), and using a transformation protocol that included acetosyringone in the infiltration medium.

Plants used in the effector screen were raised under long-day conditions (16 hr light/8 hr dark) to promote faster growth. Subsequently, when performing HR assays, we found that plants raised under short-day conditions (9 hr light, 15 hr dark) produced broader and thinner leaves that gave a more distinct and reproducible HRs. However, when *N. glutinosa* were raised solely under short-day conditions, the seedlings would sometimes develop poorly with excessively long hypocotyls. Therefore, the *N. glutinosa* used in HR assays were germinated and grown under long-day conditions for 12-14 days before being transferred to short-day conditions until being injected.

Phylogenetic analysis of the NBS region from Rpg1b has previously shown that RPM1 is not orthologous to Rpg1b, indicating that their common ability to recognize AvrB is due to convergent evolution [26]. The findings by Selote and Kachroo (2010), along with the findings from this study, reveal that not only have these two *R* genes independently evolved the ability to confer resistance to *AvrB*-expressing *P. syringae* strains, but they have also independently evolved the need for a functional *RIN4*-like protein to confer this resistance.

Through the use of virus-induced gene silencing (VIGS) of soybean *RIN4* genes, Selote and colleagues have previously determined that both *GmRIN4A* and *GmRIN4B* are required for *Rpg1b*-mediated resistance to *P. syringae* strains expressing *AvrB* [12], while *GmRIN4C* and *GmRIN4D* are not [16]. Contrary to their findings, our findings suggest that each of the individual *Gm*RIN4 proteins can be used by Rpg1b to recognize AvrB, as co-expression of any *GmRIN4* with *Rpg1b* and *AvrB* in *N. glutinosa* resulted in an HR. However, a major difference between Selote’s work and ours is that they used VIGS to silence native genes while we used transient expression to over-express foreign genes. If there is a GmRIN4 expression level threshold in soybean required for Rpg1b function, then transient overexpression would likely exceed this threshold. Therefore, it is possible that *GmRIN4C* and *GmRIN4D* could be used for *Rpg1b*-mediated resistance in soybean if they were expressed at a high enough level.

As is the case with *Rpg1b*, phylogenetic evidence and amino acid sequence comparisons indicate that *Rpg1r* and *RPM1* are not orthologous and have very little amino acid sequence similarity outside the conserved NB-ARC domain [20]. Our initial observation that co-expression of *Rpg1r* with *AvrRpm1* was sufficient to trigger HR led us to hypothesize that *Rpg1r* did not have a *GmRIN4* requirement. However, co-expression of *AvrRpt2* with *Rpg1r* and *AvrRpm1* was able to reduce the leaf collapse associated with HR. While this suggests Rpg1r requires a RIN4 homologue to detect AvrRpm1, it is not definitive. It is possible that AvrRpt2 is targeting another component of the pathway required for effector recognition or is targeting a step downstream of effector recognition. If Rpg1r does indeed use one or more *Gm*RIN4s to detect AvrRpm1, this would indicate that Rpg1r, Rpg1b, RPM1, and RPS2 have all evolved the ability to detect pathogen effectors by monitoring the status of a RIN4 homolog, suggesting that RIN4 represents a common effector target across plant species, and thus a hub guarded by multiple NB-LRR proteins.

By reconstituting the *RPS5*, *Rpg1b*, and *Rpg1r* pathways in *N. glutinosa*, we have demonstrated that this system can be used to study the molecular requirements of a variety of R proteins. With the recently published draft sequence of the *N. benthamiana* genome [27], the ability to find homologous genes involved in these pathways is as simple as performing a BLAST search. The development of the *N. glutinosa* transient system will be especially useful for performing structure/function studies on Rpg1b and Rpg1r and assessing how their ability to distinguish between AvrB and AvrRpm1 is determined.

## Materials and Methods

### Plant material

All *Nicotiana* seeds were obtained from the USDA National Plant Germplasm System Nicotiana Collection at North Carolina State University in Raleigh, NC and grown in Metro-Mix 360 potting soil. Plants used for the effector screen and MUG assay (described below) were grown in a growth chamber under long-day conditions (16 hr light/8 hr dark) at 24°C. These plants were grown for 3-4 weeks before transient transformation. *N. glutinosa* plants used in subsequent HR assays were germinated under long-day conditions for 12-14 days then transferred to short-day conditions (9 hr light/16 hr dark) for ∼2 more weeks before transformation, as these growth conditions produced leaves that were easier to infiltrate, giving HR phenotypes that were more distinct. The plants were grown at 22-24°C under both long and short-day conditions.

### *Agrobacterium*-mediated transformation

*Agrobacterium tumefaciens* strain GV3101 (pMP90) was used in all experiments. All Agrobacterium strains, except for those carrying plasmids for the transfer of *AvrRpt2* and *AvrRpt2 (C122A)*, were grown overnight at 30°C in LB media with 50 µg/mL of kanamycin and 50 µg/mL of gentamycin. The strains harboring *AvrRpt2* and *AvrRpt2 (C122A)* were selected with 5 µg/mL of tetracycline. For the effector screen, a subculture was prepared the next day by inoculating fresh LB media, plus appropriate antibiotics, with overnight culture in a 1∶10 (overnight culture∶fresh media) ratio. The subculture was incubated for approximately 5 hours at 30°C with shaking after which it was centrifuged for 8 minutes at 5000 rpm. The bacterial pellet was resuspended in sterile deionized water for infiltration. For subsequent experiments a modified procedure, optimized for the efficient transformation of *N. glutinosa*, was used. For this procedure, the overnight culture was grown until saturated (∼16 hrs) before the bacteria were pelleted and washed with 5 ml of 10 mM MgCl_2_. The pellet was then resuspended in 3 ml of a solution containing 10 mM MgCl_2_ and 100 µM acetosyringone (Sigma). The suspension was then incubated at room temperature for at least 2 hrs before being diluted to the appropriate density for injection using 10 mM MgCl_2_ and 100 µM acetosyringone. Using this modified procedure for transforming *N. glutinosa* reduced the non-specific response to the Agrobacterium and gave more consistent results. Avoiding injecting Agrobacterium strains at an OD_600_ >0.3 also reduced the non-specific response to Agrobacterium sometimes observed in *N. glutinosa*. Important factors for obtaining efficient transformation included using young plants (∼4 weeks old), avoiding the youngest and oldest leaves (typically the 3rd and 4th true leaves were injected), and using a transformation protocol that included acetosyringone in the infiltration medium.

For the effector screen and MUG assays, each Agrobacterium strain was infiltrated at an O.D._600_ of 0.3. For the HR assays in *N. glutinosa*, combinations of up to 3 strains were co-infiltrated with each strain represented at an O.D._600_ of 0.1. In these mixed inoculations the total Agrobacterium concentration remained at an O.D._600_ of 0.3. An Agrobacterium strain with an empty vector plasmid was used as filler for combinations with fewer than three strains. A 1.0 mL needleless syringe was used to infiltrate the appropriate Agrobacterium strain(s). When necessary, a needle or razor blade was used to make a hole/nick at the intended injection site to facilitate subsequent injection with the needleless syringe.

### Plasmids

The *P. syringae* effector genes *AvrPphB*, *AvrB* and *AvrRpm1* were cloned in the pTA7002 plasmid, which places the transgene under control of a dexamethasone (DEX) inducible promoter [28]. The empty vector, *RPS5*, *PBS1*, *Rpg1b*, *Rpg1r* and *GmRIN4* constructs also employed the pTA7002 vector. The *RPS5* and *PBS1* constructs contained C-terminal 5x-Myc and 3x-HA tags, respectively. The *GmRIN4* constructs contained an N-terminal 5x-Myc tag, while the *Rpg1r* construct contained a C-terminal sYFP tag. The *GUS* reporter gene was in the pCNL65 plasmid, which places the transgene under control of the cauliflower mosaic virus 35S promoter [29]. The effector genes *AvrRpt2* and *AvrRpt2 (C122A)* were also under CaMV 35S control in the pMD1 vector and each had a C-terminal 3x-HA tag [22]. Expression of DEX-inducible constructs was achieved by spraying transiently transformed plants with a solution of 50 µM dexamethasone (Sigma-Aldrich) and 0.02% Silwet-L77 (Momentive, Albany, NY) approximately 40 hours post-infiltration.

### MUG fluorometric assay

The MUG fluorometric assay for β-glucuronidase (*GUS*) activity was adapted from [30]. Unless indicated otherwise, all reagents were obtained from Sigma-Aldrich. The third youngest leaf of each plant was transiently transformed with the *GUS* reporter gene. Six leaf discs (0.6 cm in diameter) were collected from each plant approximately 40 hr post-infiltration and ground in a 1.5 mL microfuge tube with 450 µL of extraction buffer (10 mM EDTA, 0.1% Triton X-100, 0.1% sodium lauryl sarcosine, and 10 mM β-mercaptoethanol, 50 mM phosphate buffer at pH 7). Five microliters of tissue extract were added to 500 µL of 1 mM MUG reaction buffer (4-methylumbelliferone-β-D- glucuronide dissolved in extraction buffer). The reaction was incubated at 37°C and 40 µL aliquots were removed and added to 160 µL of stop buffer (0.2 M Na_2_CO_3_) in a black microtiter plate at zero time and subsequent time points. Forty microliters of each 4-methylumbelliferone (MU) standard (20-100 µM) were also added to 160 µL of stop buffer in the microtiter plate. A Bradford assay kit (Biorad) was used to normalize each sample by calculating the protein concentration according to the manufacturer's instructions.

Fluorescence and absorbance measurements were made using a Thermo Scientific Appliskan microplate reader. A 340 nm excitation filter and 500 nm emission filter were used to measure the fluorescence from the MUG assay samples. A 595 nm filter was used to measure the absorbance of the samples from the Bradford assay.

## Supporting Information

Figure S1 **Examples of leaf morphologies observed in** ***Nicotiana*** **species expressing** ***AvrB*** **or** ***AvrRpm1.*** The left image is a *N. tabacum* leaf exhibiting tissue browning from *AvrRpm1* (-) and *AvrB* (+) expression. In the center, the abaxial surface of a *N. glutinosa* leaf is exhibiting a “shiny” phenotype from both *AvrRpm1* and *AvrB* expression (a similar response was also observed in response to the *GUS* containing strain). The right image is an example of full leaf collapse in a *N. benthamiana* leaf expressing *AvrB*.
(TIF)

Figure S2 **Box and whisker plot showing quantification of transformation efficiency as determined by a MUG fluorometric assay.** The boxplot was generated from data compiled from 4 independent experiments with a total sample size of n = 28 for *N. benthamiana* and n = 29 for *N. glutinosa*. The whiskers represent minimum and maximum values of the data. The (•) symbol above the *N. benthamiana* boxplot indicates an outlying data point. Statistical significance was assessed using a two-tailed Student’s *t*-test: * indicates *P* = 0.001.
(TIF)

Figure S3 **Amino acid sequence alignment of** ***Arabidopsis*** **RIN4, the soybean RIN4s, and two putative RIN4 homologs from** ***N. benthamiana*** **.** Each AvrRpt2 RIN4 cleavage site (RCS) of *Arabidopsis* RIN4 is indicated [21].
(TIF)
