# Supplementary figures and images for: Determining the GmRIN4 Requirements of the Soybean Disease Resistance Proteins Rpg1b and Rpg1r Using a Nicotiana glutinosa-Based Agroinfiltration System
Source: PLoS One. 2014 Sep 22;9(9):e108159. doi: 10.1371/journal.pone.0108159 (PMC4171518; doi:10.1371/journal.pone.0108159)

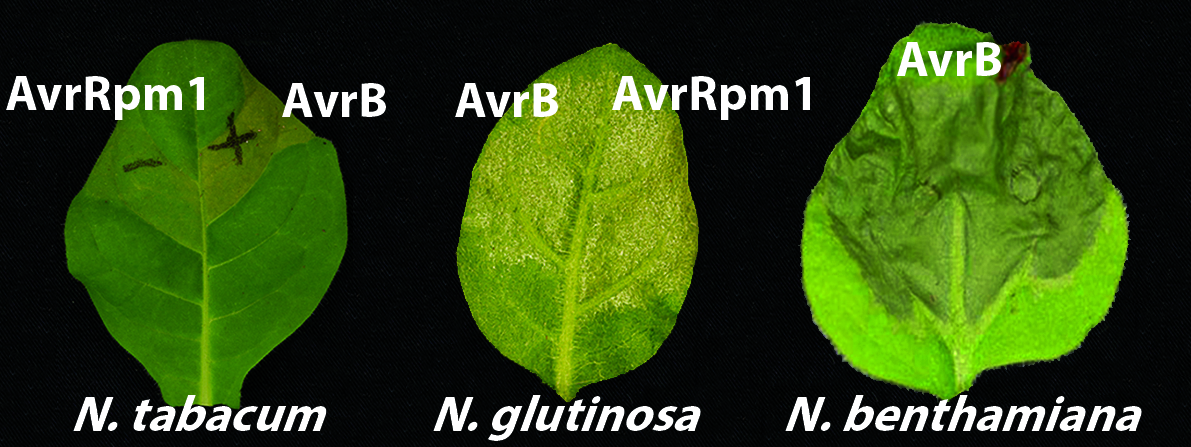

Supplement: Figure S1 — Examples of leaf morphologies observed in Nicotiana species expressing AvrB or AvrRpm1. The left image is a N. tabacum leaf exhibiting tissue browning from AvrRpm1 (-) and AvrB (+) expression. In the center, the abaxial surface of a N. glutinosa leaf is exhibiting a “shiny” phenotype from both AvrRpm1 and AvrB expression (a similar response was also observed in response to the GUS containing strain). The right image is an example of full leaf collapse in a N. benthamiana leaf expressing AvrB. (TIF) [file pone.0108159.s001.tif]

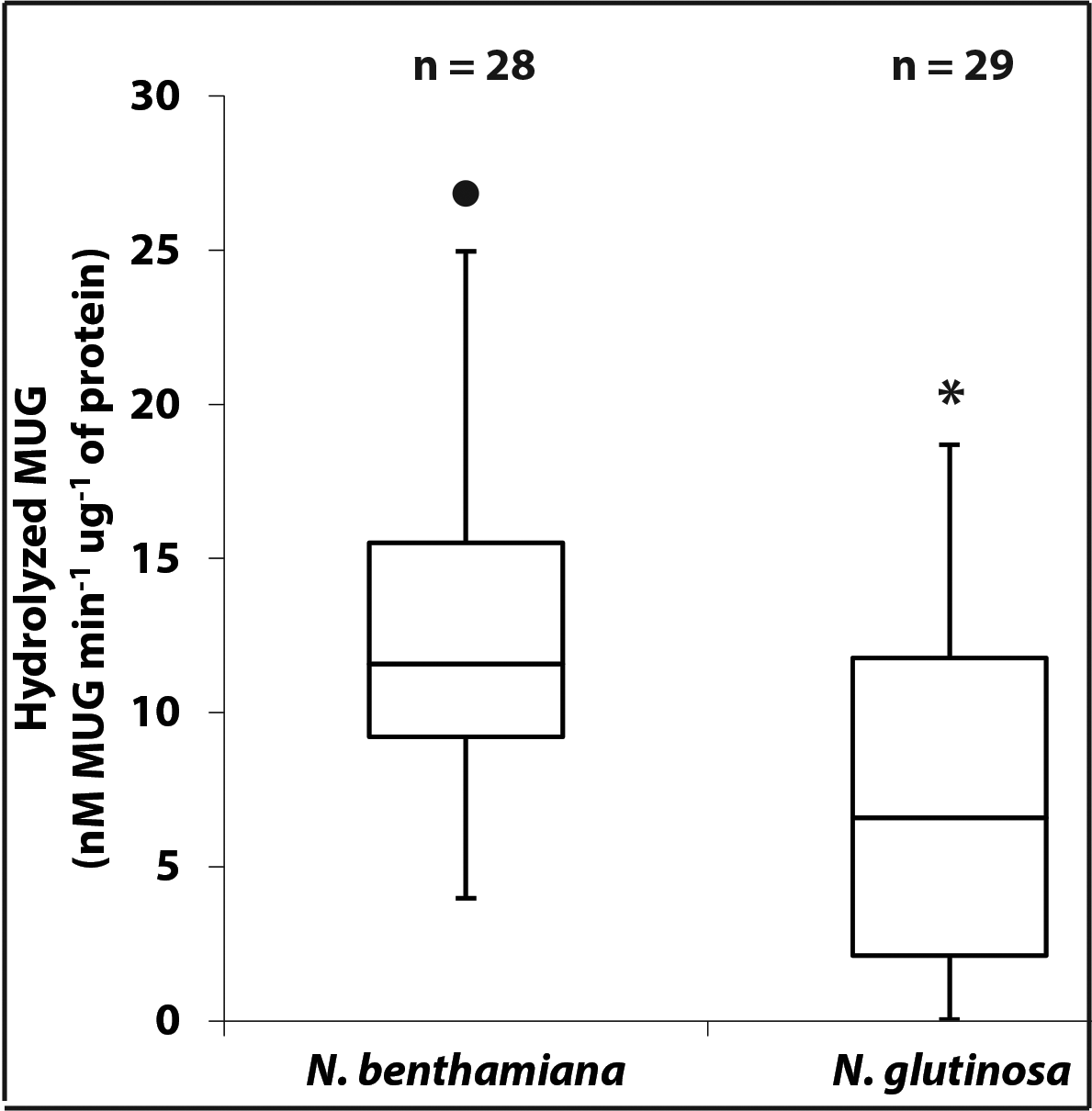

Supplement: Figure S2 — Box and whisker plot showing quantification of transformation efficiency as determined by a MUG fluorometric assay. The boxplot was generated from data compiled from 4 independent experiments with a total sample size of n = 28 for N. benthamiana and n = 29 for N. glutinosa. The whiskers represent minimum and maximum values of the data. The (•) symbol above the N. benthamiana boxplot indicates an outlying data point. Statistical significance was assessed using a two-tailed Student’s t-test: * indicates P = 0.001. (TIF) [file pone.0108159.s002.tif]

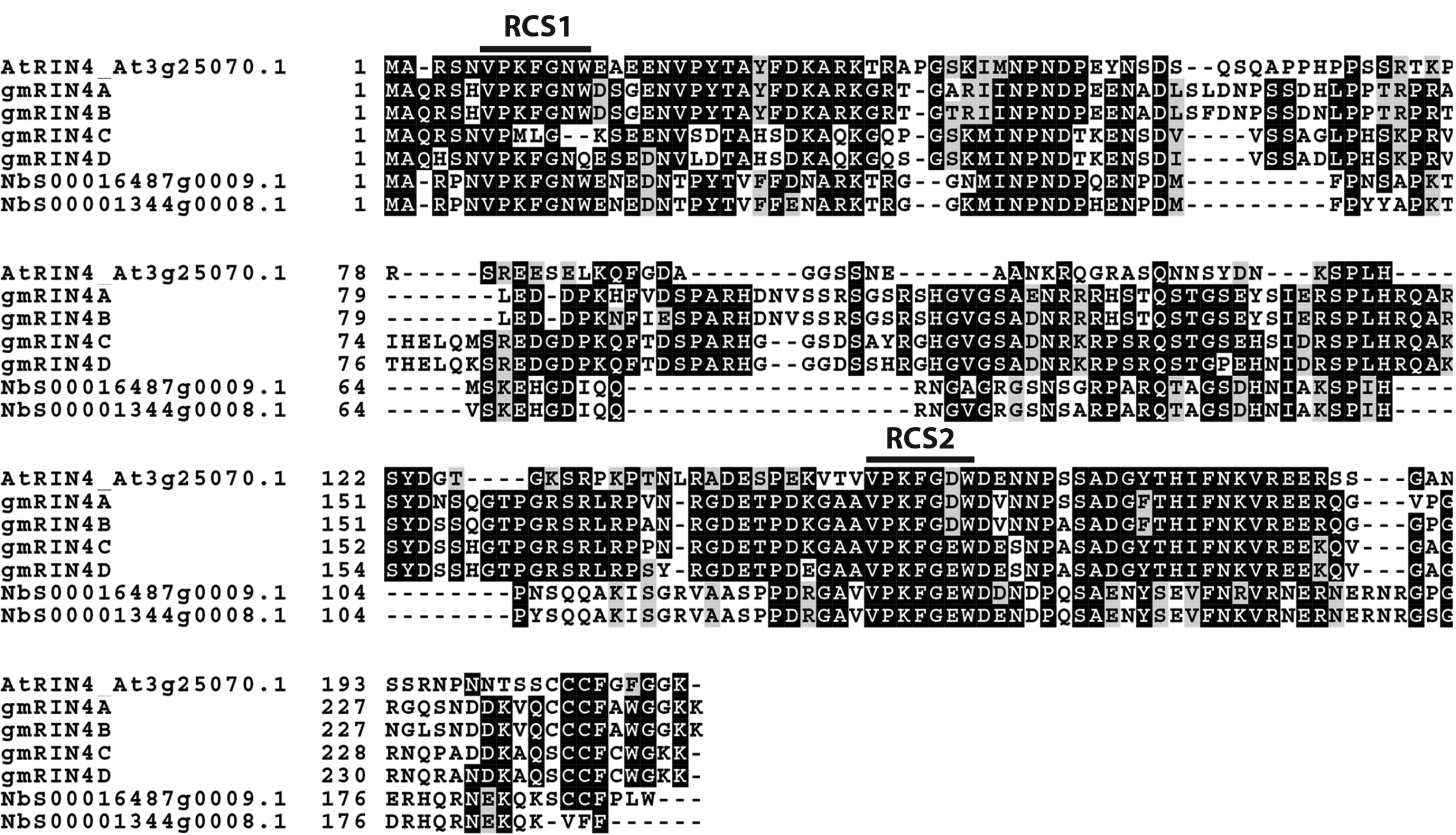

Supplement: Figure S3 — Amino acid sequence alignment of Arabidopsis RIN4, the soybean RIN4s, and two putative RIN4 homologs from N. benthamiana . Each AvrRpt2 RIN4 cleavage site (RCS) of Arabidopsis RIN4 is indicated [21]. (TIF) [file pone.0108159.s003.tif]
